# The Burden of Obesity in Cardiac Surgery: A 14 years' Follow-Up of 14.754 Patients

**DOI:** 10.1155/2024/5564810

**Published:** 2024-05-15

**Authors:** Alexander Beckmann, Maximilian Poehlmann, Patrick Mayr, Markus Krane, Johannes Boehm

**Affiliations:** ^1^Department of Cardiovascular Surgery, Institute Insure, German Heart Center Munich, School of Medicine & Health, Technical University of Munich, Lazarettstrasse 36, Munich 80636, Germany; ^2^Institute for Cardiac Anesthesiology, German Heart Center Munich, Munich, Germany; ^3^DZHK (German Center for Cardiovascular Research), Partner Site Munich Heart Alliance, Munich, Germany; ^4^Division of Cardiac Surgery, Department of Surgery, Yale School of Medicine, New Haven, CT, USA

## Abstract

**Aims:**

The prevalence of obesity is rapidly increasing during the past decades. While previous research has focused on the early outcome after cardiac surgery or specific complications, the current study covers the whole burden of obesity in the field of cardiac surgery over short term and long term. Endpoints of the study were all-cause mortality, perioperative outcome, and wound-healing disorders (WHDs).

**Methods:**

14.754 consecutive patients who underwent cardiac surgery over a 14 years' time period were analyzed. BMI classifications were used according to the WHO definition.

**Results:**

Mean survival was 11.95 years ± 0.1; CI 95% [12.04–12.14]. After adjustment for clinical baseline characteristics, obesity classes' I–III (obesity) did not affect 30-day mortality or all-cause mortality during the whole observational period. After adjustment for known risk factors, the risk for WHDs doubled at least in obesity patients as follows: obesity I (OR = 2.06; CI 95% [1.7–2.5]; *p* < 0.0001), obesity II (OR = 2.5; CI 95% [1.83–3.41]; *p* < 0.0001), and obesity III (OR = 4.12; CI 95% [2.52–6.74]; *p* < 0.0001). The same applies to the risk for sternal reconstruction that is substantially elevated in obesity I (OR = 2.23; CI 95% [1.75–2.83]; *p* < 0.0001), obesity II (OR = 2.81; CI 95% [1.91–4.13]; *p* < 0.0001), and obesity III (OR = 2.31; CI 95% [1.08–4.97]; *p*=0.03). No significant correlation could be found between obesity and major adverse events in the perioperative course like renal failure, ventilation >24 h, re-exploration, or cerebrovascular events.

**Conclusions:**

Cardiac surgery is safe in obesity as short- and long-term mortality are not increased, and major adverse events during the perioperative course are similar to control patients. The burden of obesity lies in substantially increased rates of wound-healing disorders and sternal reconstructions.

## 1. Introduction

According to the World Health Organization (WHO), the worldwide prevalence of obesity nearly tripled between 1975 and 2016. In 2016, nearly 39% of men over 18 years were overweight with a Body Mass Index (BMI) of 25–30 and 13% were obese with a BMI >30 (the WHO European Regional Obesity Report 2022, Copenhagen). Regarding the worldwide trends in underweight and obesity from 1990 to 2022, the combined burden of underweight and obesity has increased in most countries, driven by an increase in obesity [1]. Obesity is a well-established risk factor for multiple cardiovascular diseases, such as coronary disease, coronary death, and congestive heart failure [2]. It increases the likelihood of occurrence and severity of cardiovascular risk factors, including dyslipidemia, diabetes, hypertension, and sleep disorders [2, 3]. The increasing prevalence of obesity makes it one of the most critical public health problems worldwide, with enormous implications for treatment strategies and costs.

As obese patients are at higher risks for cardiovascular diseases, consequently the number of obese patients needing cardiac surgery will increase. Previous studies showed that the impact of obesity on perioperative mortality and major in-hospital adverse events as well as on early outcome after cardiac surgery is variable [4–10]. Variable results are also reported for subgroups like valve surgeries [5, 11] or patients with acute type A aortic dissections [12, 13]. In contrast, extreme obesity with a BMI >40 had significant increase in length of stay, rate of renal failure with necessity of renal replacement therapy, or prolonged ventilation compared to nonobese patients [14]. No difference was found in the rate of stroke [13–19]. These findings may lead to increased risk-adjusted hospital costs that might be up to 17% higher in obese patients undergoing cardiac surgery [9].

Studies analyzing the long-term outcome are lacking, while previous research has mainly focused on the early outcome after cardiac surgery. While previous research has focused on the early outcome after cardiac surgery or specific complications, the current study covers the whole burden of obesity in the field of cardiac surgery over short term and long term.

## 2. Methods

### 2.1. Study Design

This study presents a large single-center, retrospective analysis of all patients who underwent cardiac surgery in the German Heart Center Munich using cardiopulmonary bypass between 2002 and 2017. Data were obtained from an ongoing quality assessment program. All medical reports, including operative protocols, in-hospital, and outpatient notes, were reviewed.

BMI classifications were used according to the WHO definition as follows: underweight: <18.5 kg/m^2^, normal weight: 18.5–25 kg/m^2^, overweight: 25–30 kg/m^2^, and obese: >30 kg/m^2^. Obesity is divided into the following categories: class 1 (BMI of 30–34.9 kg/m^2^), class 2 (BMI of 35–39.9 kg/m^2^), and class 3 (BMI >40 kg/m^2^).

### 2.2. Definitions

- Survival: all-cause mortality was measured. Cox regression analysis was applied for further risk stratification for the total observational period and for the assessment of 30-day mortality, logistic regression analyses were used.
- Wound-healing disorder (WHD): every sternal wound-healing disorder after cardiac surgery requiring surgical care was counted as WHD, including sternal reconstruction. The inflammatory status of the wound was not additionally classified.
- Re-exploration: re-exploration due to bleeding was defined as a life-threatening, major or minor bleeding according to the BARC criteria, which requires surgical intervention.
- Long-term ventilation: long-term ventilation was defined as any necessity for postoperative ventilation >24 h.
- Renal replacement therapy: all kind of life-supporting treatments for renal failure applied intermittently or continuously using extracorporeal methods.
- Cerebrovascular event: episode of focal or global neurological deficit ≥24 h or <24 h if available neuroimaging documents with at least one of the following: change in the level of consciousness, hemiplegia, hemiparesis, aphasia, or other neurological signs or symptoms according to the VARC-3 criteria.

### 2.3. Statistical Analysis

Statistical analysis was assessed by using IBM SPSS Statistics 28.0 software (IBM Corp, Armonk, NY USA) and NCSS 20 Statistical software. Data are presented as the mean ± standard deviation for continuous variables and number (%) for categorical variables. For mean values, analysis of variance (ANOVA) was used and Chi-square tests for categorical variables as appropriate. Pearson correlation coefficients were calculated for the evaluation of bivariate correlations. Multiple regression analyses were used to measure the impact of BMI classes on clinical outcome parameters. Survival rates were calculated using Kaplan–Meier methods. *p* values were two sided and subject to a significance level of 5%.

## 3. Results

14.754 consecutive patients who underwent cardiac surgery using cardiopulmonary bypass over 14 years' period were included to the following analysis. Demographics, intraoperative, and postoperative data are provided in Table 1.

### 3.1. Demographics

The vast majority of patients had a normal BMI or were overweight (*n* = 11.524; 78.1%), whereas only 109 (0.7%) patients were cachectic. There were 3.121 (21.1%) patients with obesity I–III, and 152 (1.0%) patients suffered from obesity III. Demographic data showed significant differences regarding age, gender, the prevalence of sinus rhythm, or creatinine levels. Cachectic patients were younger (62.4 years compared to 65.5 years in the total cohort) as well as patients with obesity III (63.1 years). No differences could be observed regarding LV-function (Table 1).

The BMI classes differed substantially about comorbidities. Not surprisingly, patients with obesity suffered substantially more from diabetes with 34.0% in obesity I, 43.3% in obesity II, and 50.7% in obesity III compared to just 22.4% of patients of the total cohort and 8.9% of cachectic patients (*p* < 0.001; Table 1). Similar patterns were found for arterial hypertension and pulmonary obstruction. Notably, 11.0% of the patients with cachexia had a stroke in history compared to the total cohort with 4.1% (*p*=0.014), and they had also more malignancies (12.8% versus 6.0%, respectively) but this finding did not reach significance (*p*=0.077; Table 1).

Patients with cachexia or normal BMI were less likely to undergo CABG (16.5% and 27.3% versus 35.8% of the total cohort, respectively; *p* < 0.001) but had substantially more valve surgery (49.5% and 38.6% versus 31.7% of the total cohort; *p* < 0.001). The CPB time, however, as a surrogate parameter for the complexity of the surgical procedure did not differ between the BMI classes (Table 1).

### 3.2. Survival

**Figure 1:**
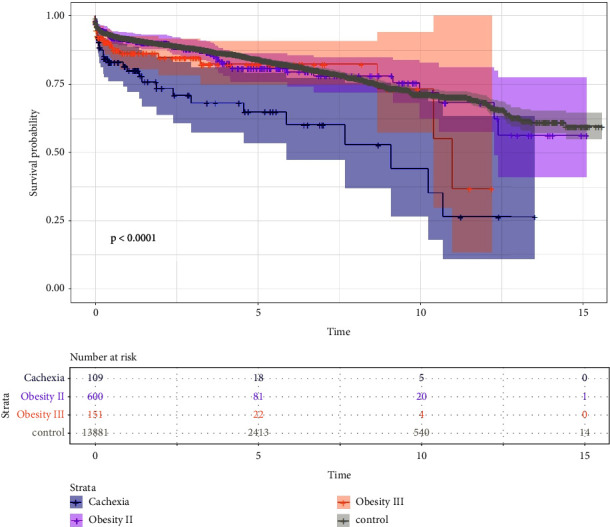
Kaplan–Meier estimates for survival.

**Figure 2:**
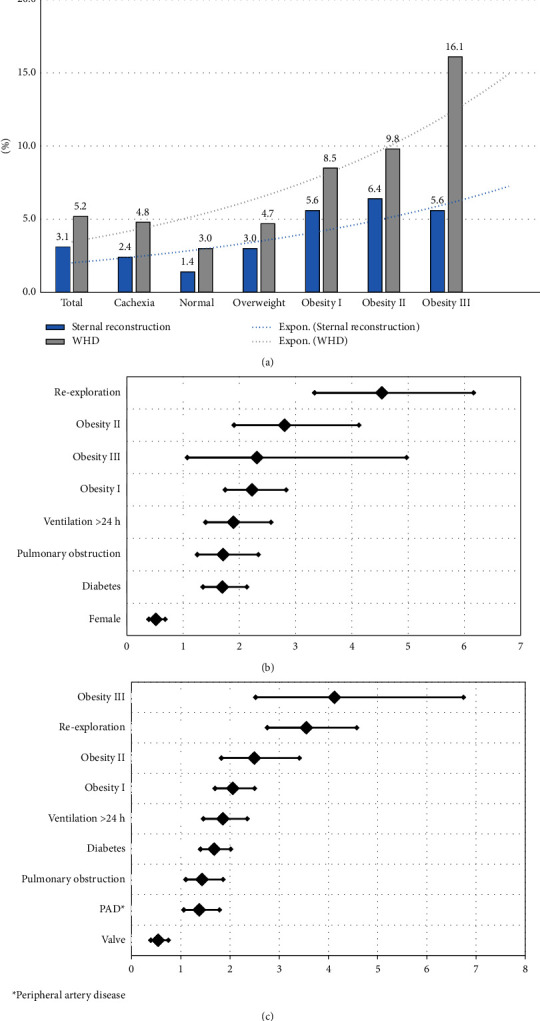
(a) Outcome of wound healing according to BMI classes. (b) Odds ratios of the influencing factors of sternal reconstruction. (c) Odds ratios of the influencing factors of a WHD.

Mean survival was 11.95 years ± 0.1; CI 95% [12.04–12.14]. Survival data differed between the groups for all-cause mortality (Table 2(a)) but not for 30-day mortality (Table 2(b)). Kaplan–Meier survival estimates and survival probability plot show significant impaired survival outcome for cachectic patients (Figures 1 and 2(a)) but not for obesity I–III. Cox regression analysis found cachexia as the independent risk factor for mortality (OR = 2.733; CI 95% [1.874–3.988]; *p* < 0.0001), independent from known risk factors such as age, creatinine, LV-function, diabetes, CPB time, postoperative renal failure, long-term ventilation, re-exploration, or cerebrovascular events (Table 2(a)). Furthermore, no association between BMI classes and 30-day mortality was observed although cachexia had substantially higher odds for mortality but this finding did not reach significance (OR = 2.162; CI 95% [0.941–4.967]; *p*=0.069).

### 3.3. Wound Healing

Sternotomy was performed in 13.670 patients (92.7%) and these patients were further analyzed with regards to wound healing. Obesity class I–III had their most impressive impact on the outcome of wound healing.  Figure 2(a) demonstrates significantly higher percentages of WHD or sternal reconstruction for patients with obesity I–III compared with the total cohort. Multiple logistic regression analyses for the risk of sternal reconstruction (Table 3(a)) and WHD (Table 3(b)) have been performed.

The risk of needing sternal reconstruction in obesity patients is at least twice as high as in the control group as follows: Obesity I (OR = 2.23; CI 95% [1.75–2.83]; *p* < 0.0001), obesity II (OR = 2.81; CI 95% [1.091–4.13]; *p* < 0.0001), and obesity III (OR = 2.31; CI 95% [1.08–4.97]; *p*=0.03). The same applies to WHDs as follows: obesity I (OR = 2.06; CI 95% [1.7–2.5]; *p* < 0.0001), obesity II (OR = 2.5; CI 95% [1.83–3.41]; *p* < 0.0001), and obesity III (OR = 4.12; CI 95% [2.52–6.74]; *p* < 0.0001).

Factors that had a significant impact on sternal reconstructions or WHDs were further analyzed, and the forest plots of their odds ratios are given in Figures 2(b) and 2(c), respectively. Notably, the highest odd for sternal reconstruction depicts re-exploration during the postoperative period (OR = 4.535; CI 95% [3.337–6.156]; *p* < 0.001) followed by obesity I–III. The highest odds for WHD, however, was observed in obesity III (OR = 4.123; CI 95% [2.521–6.743]; *p* < 0.001) just ahead of re-exploration and obesity I-II.

### 3.4. Perioperative Outcome

Analyzing major adverse events during the short-term, multiple logistic regression analyses were performed. Demographic data as well as comorbidities and surgical parameters were included in the multiple logistic regression analysis. Neither cachexia nor obesity I–III were associated with an increased risk for renal replacement therapy (Table 3(c)), long-term ventilation (Table 3(d)), re-exploration (Table 3(e)), or cerebrovascular events (Table 3(f)). Unexpectedly, cachexia was found to be a substantial higher risk for long-term ventilation postoperatively (OR = 2.812; CI 95% [1.695–4.668]; *p* < 0.001; Table 3(d)).

## 4. Discussion

Obesity is a well-established risk factor for cardiovascular disease, diabetes mellitus, and hypertension [20] and is likely to play an increasingly important role in cardiac surgery in the future.

### 4.1. Survival and Perioperative Outcome

Little is known about the effect of obesity on long-term outcome after cardiac surgery with a follow-up of more than one year [8]. Former research suggested that patients with obesity or at least overweight might have a survival benefit, culminating in the term “obesity paradox” that states that at least mildly obese patients with heart failure might have better clinical outcome than expected [4, 7, 21, 22]. Moreover, Zhang et al. suggested a survival benefit of patients with BMI>30 kg/m^2^ after cardiac surgery in a subgroup of elderly patients [23]. The effects on survival, however, appeared to be small or almost zero and are controversial [8, 9, 11, 19]. The current analysis did not find any survival benefit in obesity patients, neither for the 30-day mortality nor for the long-term outcome. Unlike former research, the current study analyzes a long observational period of 15 years (mean survival of 4.360 ± 39 days, Table 1), with no effects of obesity I–III on survival.

No significant associations could be observed with major adverse events during the postoperative period (Tables 3(c)–3(f)). These findings limit the suggestions of former research that reported an increased perioperative morbidity in obesity patients [14, 24]. The current study did not find strong effects of obesity I–III on renal failure but provides some indication for a possible, small risk in obesity III (OR = 1.623; CI 95% [0.949–2.776]; *p*=0.077; Table 3(c)). The same applies for prolonged ventilation (Table 3(d)). Furthermore, obesity I–III did not come out as a risk factor for cerebrovascular events (Table 3(f)), which is in line with former research [6]. Notably, the necessity for re-exploration or the length of CPB times was not increased in obese patients, which might have been assumed given the technically more difficult operational site. Taken together, the impact of obesity I–III on perioperative morbidity is either very small or as the current study suggests, simply does not exist.

### 4.2. Cachexia

We found that indeed cachexia was an independent risk factor for all-cause mortality (OR = 2.733; CI 95% [1.874–3.988]; *p* < 0.0001; Table 2(a)) as well as a risk factor for prolonged ventilation in the postoperative period (OR = 2.812; CI 95% [1.695–4.668]; *p* < 0.0001; Table 3(d)). As we could rule out malignancies as the main reason for this finding (Table 1), cachexia might represent an unspecific marker for the end stage of the underlying cardiac disease. Moreover, the impaired survival of cachectic patients may explain some of the beneficial effects of obesity on survival. If cachectic patients were not analyzed separately in the previous studies, the catastrophic effects of cachexia may have biased the results in favor of obese patients, thus misrepresenting an advantage for obesity.

### 4.3. The Burden of Obesity

The current study suggests that the main disadvantage of obesity in patients undergoing cardiac surgery lies in the impaired outcome of wound healing after sternotomy. Former studies showed that obesity is independently associated with an increased risk of postoperative sternal wound infection [16, 25], especially with a BMI over 30 kg/m^2^ [26]. In order to study a real world situation, we counted any surgical intervention due to wound healing as WHD regardless from their infective state. The CDC classification focuses on the infective state [27] but might lose WHD that are noninfective but require surgery nonetheless. The specific approach of the current study demonstrates slightly elevated WHDs' numbers but shows a real world situation of obese patients and gives so a broader perspective to the study. Furthermore, we analyzed the subgroup of patients that required sternal reconstruction. Taken together, every additional point of BMI worsens the outcome of wound healing (Figure 2(a)), and the risk for sternal reconstructions or WHDs doubled at least in obesity patients (Tables 3(a) and 3(b)).

### 4.4. Implications of Obesity in Cardiac Surgery

As cardiac surgery will be confronted with more obese patients in the foreseeable future, strategies to reduce WHDs are urgently needed. Minimally invasive techniques that do not require conventional sternotomy seem to be the most promising options in cardiac surgery in obese patients: In mitral valve surgery, lateral mini thoracotomy becomes more and more common[28], and catheter techniques [29] or robotic surgery are emerging [30]. In aortic valve disease, the ongoing discussion about the indications between transcatheter aortic valve implantations and conventional approaches via sternotomy [31] should be[32] extended[33] and obese[34] patients should be treated.

## 5. Conclusions

There is no increased risk of short- or long-term mortality after cardiac surgery in overweight patients compared with normal-weight patients. In addition, the major adverse events during the perioperative course are similar to those in normal-weight patients. The major adverse effect of obesity is the significantly increased rate of wound healing disorders and sternal reconstruction. Unexpectedly, patients with cachexia without apparent oncologic disease have a significantly increased risk of both the occurrence of perioperative complications and increased short- and long-term mortality.

## Figures and Tables

**Table 1:** Patients characteristics.

	Total	Cachexia	Normal	Overweight	Obesity I	Obesity II	Obesity III	*p* value
*n* (%)	14.754	109 (0.7%)	4.553 (30.9%)	6.971 (47.2%)	2.369 (16.0%)	600 (4.1%)	152 (1.0%)	
Demographics								
BMI (kg/m^2^)	27.1 ± 4.3	17.3 ± 1.0	22.96 ± 1.6	27.2 ± 1.4	31.8 ± 1.4	36.9 ± 1.4	43.2 ± 3.1	
Height (cm)	171.3 ± 8.8	168.3 ± 10.5	171.5 ± 9.2	172.0 ± 8.4	170.5 ± 8.8	168.3 ± 8.9	165.9 ± 9.0	
Weight (kg)	79.7 ± 14.3	49.2 ± 6.8	67.8 ± 8.9	80.6 ± 8.7	92.7 ± 10.0	104.8 ± 11.7	119.4 ± 16.0	
Age (years)	65.5 ± 11.4	62.4 ± 16.7	64.6 ± 12.8	66.00 ± 10.9	66.06 ± 10.21	65.56 ± 9.5	63.06 ± 10.3	<0.001
Female (%)	4.456 (30.2%)	32 (29.4%)	1.606 (35.3%)	1.800 (25.8%)	669 (28.2%)	226 (37.7%)	78 (51.3%)	<0.001
Sinus rhythm (%)	12.155 (82.4%)	79 (72.5%)	3.641 (80.0%)	5.846 (83.9%)	1.975 (83.4%)	495 (82.5%)	119 (78.3%)	<0.001
Creatinine (mg/dl)	1.03 ± 0.36	0.86 ± 0.31	1.01 ± 0.37	1.03 ± 0.35	1.05 ± 0.36	1.04 ± 0.32	1.17 ± 0.5	<0.001
Reduced LV-EF (%)^*∗*^	3.281 (22.2%)	21 (19.3%)	954 (21.0%)	1.595 (22.9%)	535 (22.6%)	134 (22.3%)	42 (27.7%)	0.098
Severely reduced LV-EF (%)^*∗∗*^	631 (4.3%)	4 (3.7%)	191 (4.2%)	296 (4.2%)	103 (4.3%)	27 (4.5%)	10 (6.6%)	0.812
Comorbidities								
Diabetes mellitus (%)	3.122 (22.4%)	9 (8.9%)	612 (14.3%)	1.418 (21.5%)	763 (34.0%)	246 (43.3%)	74 (50.7%)	<0.001
Arterial hypertension (%)	11.062 (76.5%)	46 (44.7%)	2.934 (66.3%)	5.418 (79.1%)	2.003 (85.6%)	530 (88.8%)	131 (87.9%)	<0.001
Pulmonary obstruction (%)	1.174 (8.0%)	9 (8.3%)	330 (7.2%)	523 (7.5%)	210 (8.9%)	82 (13.7%)	20 (13.2%)	<0.001
Pulmonal hypertension (%)	2.097 (14.2%)	25 (22.9%)	768 (16.9%)	860 (12.3%)	320 (13.5%)	87 (14.5%)	37 (24.3%)	<0.001
Peripheral artery disease (%)	1.005 (6.8%)	7 (6.4%)	292 (6.4%)	470 (6.7%)	186 (7.9%)	40 (6.7%)	10 (6.6%)	0.382
Stroke in history (%)	607 (4.1%)	12 (11.0%)	192 (4.2%)	271 (3.9%)	100 (4.2%)	25 (4.2%)	7 (4.6%)	0.014
Malignancy (%)	886 (6.0%)	14 (12.8%)	272 (6.0%)	407 (5.8%)	143 (6.0%)	40 (6.7%)	10 (6.6%)	0.077
Intraoperative parameter								
CABG (%)^*∗∗∗*^	5.287 (35.8%)	18 (16.5%)	1.242 (27.3%)	2.739 (39.3%)	995 (42.0%)	238 (39.7%)	55 (36.2%)	<0.001
Valve	4.671 (31.7%)	54 (49.5%)	1.758 (38.6%)	2.029 (29.1%)	604 (25.5%)	172 (28.7%)	53 (34.9%)	<0.001
CABG + valve	2.135 (14.5%)	8 (7.3%)	627 (13.8%)	1.005 (14.4%)	383 (16.2%)	92 (15.3%)	20 (13.2%)	0.030
Others	2.661 (18.0%)	29 (26.6%)	926 (20.3%)	1198 (17.2%)	386 (16.3%)	98 (16.3%)	24 (15.8%)	<0.001
CPB time (minutes)^†^	111 ± 49	115 ± 47	114 ± 53	110 ± 48	112 ± 48	110 ± 46	113 ± 46	0.843
Short–term outcome								
Renal replacement therapy	1.017 (6.9%)	9 (8.3%)	319 (7.0%)	442 (6.3%)	176 (7.4%)	50 (8.3%)	21 (13.8%)	0.003
Ventilation >24 h	1.543 (10.5%)	25 (22.9%)	478 (10.5%)	689 (9.9%)	252 (10.6%)	74 (12.3%)	25 (16.4%)	<0.001
Cerebrovascular event	520 (3.6%)	7 (6.5%)	180 (4.0%)	239 (3.5%)	76 (3.2%)	14 (2.4%)	4 (2.7%)	0.105
Re-exploration (bleeding)	811 (5.5%)	10 (9.2)	282 (6.2%)	361 (5.2%)	126 (5.3%)	23 (3.8)	9 (5.9)	0.037
Long-term outcome								
All-cause mortality	1.729 (11.7%)	30 (27.5)	558 (12.3%)	769 (11.0%)	279 (11.8%)	70 (11.7)	23 (15.2)	<0.0010
30-day mortality	645 (4.4%)	8 (7.3)	209 (4.6%)	290 (4.2)	99 (4.2%)	28 (4.7)	11 (7.2)	0.230
Mean survival (days)	4.360 ± 39	2.740 ± 296	4.245 ± 61	4.382 ± 53	4.362 ± 88	4.201 ± 171	3.401 ± 198	<0.0010
Wound healing								
*n* (%)	12.611	83 (0.7%)				560 (4.4%)	143 (1.1%)	
Sternal reconstruction	394 (3.1%)	2 (2.4%)	51 (1.4%)	178 (3.0%)	119 (5.6%)	36 (6.4%)	8 (5.6%)	<0.0010
WHD^††^	654 (5.2%)	4 (4.8%)	109 (3.0%)	283 (4.7%)	180 (8.5%)	55 (9.8%)	23 (16.1%)	<0.0010

^*∗*^Left ventricular ejection fraction: 31–50%; ^*∗∗*^left ventricular ejection fraction ≤30%; ^*∗∗∗*^coronary artery bypass grafting; ^†^cardiopulmonary bypass time; ^††^wound-healing disorder.

**Table 2:** (a) Cox regression analysis for all-cause mortality. (b) Logistic regression analysis for 30-day mortality.

	*B*	OR	Lower CI	Upper CI	*p* value
(a)					
Age	0.039	1.040	1.034	1.046	<0.0001
Female	0.093	1.097	0.984	1.224	0.096
Sinus rhythm	−0.114	0.892	0.793	1.005	0.060
Creatinine	0.220	1.246	1.131	1.373	<0.0001
Reduced LV-EF^*∗*^	0.200	1.221	1.089	1.368	0.001
Severely reduced LV-EF^*∗∗*^	0.436	1.546	1.284	1.862	<0.0001
BMI category					
Cachexia	1.006	2.733	1.874	3.988	<0.0001
Obesity I	−0.075	0.928	0.807	1.066	0.291
Obesity II	0.025	1.025	0.795	1.323	0.846
Obesity III	0.228	1.256	0.820	1.924	0.294
Comorbidities					
Diabetes mellitus	0.250	1.284	1.143	1.442	<0.0001
Arterial hypertension	−0.083	0.920	0.811	1.045	0.200
Pulmonary obstruction	0.345	1.412	1.221	1.632	<0.0001
Pulmonal hypertension	−0.030	0.971	0.850	1.109	0.660
Peripheral artery disease	0.383	1.466	1.255	1.714	<0.0001
Stroke in history	0.279	1.322	1.088	1.606	0.005
Malignancy	0.184	1.202	1.007	1.435	0.042
Intraoperative data					
CABG^*∗∗∗*^	−0.173	0.841	0.714	0.991	0.039
Valve	−0.095	0.909	0.785	1.053	0.205
CABG + valve	−0.204	0.815	0.693	0.959	0.013
Cardiopulmonary bypass time	0.003	1.003	1.002	1.004	<0.0001
Postoperative course					
Renal replacement therapy	1.525	4.594	3.996	5.281	<0.0001
Ventilation <24 h	0.474	1.606	1.408	1.833	<0.0001
Re-exploration (bleeding)	0.352	1.421	1.220	1.656	<0.0001
Cerebrovascular event	0.593	1.810	1.543	2.123	<0.0001
Sternal reconstruction	−0.012	0.988	0.766	1.275	0.927

(b)					
Age	0.027	1.027	1.016	1.038	<0.0001
Female	0.454	1.574	1.268	1.953	<0.0001
Sinus rhythm	−0.010	0.990	0.780	1.256	0.932
Creatinine	0.212	1.236	1.014	1.507	0.036
Reduced LV-EF^*∗*^	0.234	1.263	1.006	1.587	0.044
Severely reduced LV-EF^*∗∗*^	0.456	1.578	1.079	2.310	0.019
BMI category					
Cachexia	0.771	2.162	0.941	4.967	0.069
Obesity I	−0.232	0.793	0.596	1.056	0.112
Obesity II	−0.045	0.956	0.579	1.579	0.860
Obesity III	0.112	1.119	0.522	2.399	0.773
Comorbidities					
Diabetes mellitus	0.153	1.166	0.920	1.477	0.204
Arterial hypertension	−0.188	0.828	0.641	1.071	0.150
Pulmonary obstruction	0.225	1.253	0.915	1.715	0.159
Pulmonal hypertension	−0.194	0.823	0.633	1.071	0.148
Peripheral artery disease	0.336	1.400	1.010	1.939	0.043
Stroke in history	0.386	1.471	0.990	2.187	0.056
Malignancy	−0.248	0.780	0.529	1.150	0.211
Intraoperative data					
CABG^*∗∗∗*^	−0.071	0.931	0.675	1.284	0.664
Valve	−0.224	0.799	0.597	1.070	0.132
CABG + valve	−0.266	0.767	0.557	1.055	0.103
Cardiopulmonary bypass time	0.008	1.008	1.006	1.009	<0.0001
Postoperative course					
Renal replacement therapy	2.576	13.143	10.350	16.690	<0.0001
Ventilation <24 h	0.641	1.898	1.502	2.398	<0.0001
Re-exploration (bleeding)	0.524	1.689	1.284	2.222	<0.0001
Cerebrovascular event	0.699	2.011	1.484	2.724	<0.0001
Constant	−7.147	0.001			<0.0001

^*∗*^Left ventricular ejection fraction: 31–50%; ^*∗∗*^left ventricular ejection fraction ≤30%; ^*∗∗∗*^coronary artery bypass grafting.

**Table 3:** (a) Multiple logistic regression analysis for sternal reconstruction. (b) Multiple logistic regression analysis for wound-healing disorders. (c) Multiple logistic regression analysis for renal replacement therapy. (d) Multiple logistic regression analysis for ventilation >24 h. (e) Multiple logistic regression analysis for re-exploration. (f) Multiple logistic regression analysis for cerebrovascular events.

	*B*	OR	Lower CI	Upper CI	*p* value
(a)					
Age	0.006	1.006	0.994	1.018	0.323
Gender	−0.703	0.495	0.369	0.663	<0.0001
Sinus rhythm	−0.060	0.942	0.699	1.269	0.692
Creatinine	0.188	1.207	0.924	1.575	0.167
Reduced LV-EF^*∗*^	0.141	1.152	0.905	1.466	0.250
Severely reduced LV-EF^*∗∗*^	0.368	1.445	0.966	2.161	0.073
BMI category					
Cachexia	0.306	1.359	0.300	6.142	0.691
Obesity I	0.800	2.226	1.749	2.833	<0.0001
Obesity II	1.031	2.805	1.907	4.126	<0.0001
Obesity III	0.838	2.313	1.076	4.973	0.032
Comorbidities					
Diabetes mellitus	0.530	1.699	1.353	2.134	<0.0001
Arterial hypertension	0.118	1.125	0.826	1.533	0.455
Pulmonary obstruction	0.538	1.712	1.254	2.337	0.001
Pulmonal hypertension	−0.219	0.803	0.559	1.155	0.237
Peripheral artery disease	0.306	1.359	0.989	1.867	0.059
Stroke in history	−0.437	0.646	0.364	1.147	0.136
Malignancy	0.079	1.082	0.699	1.676	0.723
Introperative data					
CABG^*∗∗∗*^	0.344	1.410	0.984	2.022	0.061
Valve	−0.619	0.538	0.342	0.848	0.008
CABG + valve	0.151	1.163	0.791	1.709	0.444
Cardiopulmonary bypass time	−0.003	0.997	0.995	1.000	0.051
Postoperative course					
Renal replacement therapy	−0.203	0.816	0.552	1.207	0.309
Ventilation <24 h	0.639	1.895	1.401	2.564	<0.0001
Re-exploration (bleeding)	1.512	4.535	3.337	6.165	<0.0001
Cerebrovascular event	−0.033	0.967	0.590	1.587	0.895
Constant	−4.520	0.011			<0.0001

(b)					
Age	−0.003	0.997	0.988	1.006	0.578
Gender	0.008	1.008	0.830	1.224	0.937
Sinus rhythm	−0.083	0.921	0.732	1.158	0.480
Creatinine	−0.033	0.967	0.770	1.214	0.775
Reduced LV-EF^*∗*^	0.097	1.102	0.909	1.334	0.323
Severely reduced LV-EF^*∗∗*^	0.178	1.194	0.846	1.687	0.313
BMI category					
Cachexia	0.278	1.321	0.462	3.773	0.603
Obesity I	0.721	2.057	1.695	2.498	<0.0001
Obesity II	0.914	2.495	1.826	3.409	<0.0001
Obesity III	1.417	4.123	2.521	6.743	<0.0001
Comorbidities					
Diabetes mellitus	0.518	1.679	1.400	2.014	<0.0001
Arterial hypertension	0.097	1.101	0.870	1.395	0.423
Pulmonary obstruction	0.358	1.431	1.102	1.858	0.007
Pulmonal hypertension	−0.182	0.833	0.635	1.093	0.187
Peripheral artery disease	0.319	1.376	1.061	1.785	0.016
Stroke in history	−0.249	0.780	0.512	1.189	0.248
Malignancy	0.116	1.123	0.802	1.571	0.500
Introperative data					
CABG^*∗∗∗*^	0.233	1.262	0.965	1.650	0.089
Valve	−0.618	0.539	0.390	0.747	<0.0001
CABG + valve	0.140	1.151	0.865	1.530	0.334
Cardiopulmonary bypass time	0.000	1.000	0.998	1.002	0.714
Postoperative course					
Renal replacement therapy	0.134	1.144	0.854	1.532	0.368
Ventilation <24 h	0.617	1.853	1.461	2.351	<0.0001
Re-exploration (bleeding)	1.267	3.552	2.758	4.574	<0.0001
Cerebrovascular event	0.004	1.004	0.688	1.465	0.985
Constant	−3.417	0.033			<0.0001

(c)					
Age	0.040	1.041	1.032	1.050	<0.0001
Gender	0.453	1.574	1.342	1.846	<0.0001
Sinus rhythm	−0.417	0.659	0.557	0.778	<0.0001
Creatinine	1.605	4.977	4.269	5.801	<0.0001
Reduced LV-EF^*∗*^	0.183	1.201	1.017	1.419	0.031
Severely reduced LV-EF^*∗∗*^	0.612	1.845	1.401	2.429	<0.0001
BMI category					
Cachexia	0.383	1.466	0.674	3.187	0.334
Obesity I	0.050	1.052	0.865	1.279	0.614
Obesity II	0.254	1.289	0.920	1.807	0.140
Obesity III	0.484	1.623	0.949	2.776	0.077
Comorbidities					
Diabetes mellitus	0.537	1.711	1.449	2.021	<0.0001
Arterial hypertension	−0.121	0.886	0.733	1.072	0.213
Pulmonary obstruction	0.082	1.085	0.862	1.365	0.486
Pulmonal hypertension	0.369	1.446	1.210	1.728	<0.0001
Peripheral artery disease	0.363	1.438	1.133	1.826	0.003
Stroke in history	0.157	1.170	0.860	1.590	0.317
Malignancy	0.263	1.301	1.006	1.682	0.045
Introperative data					
CABG^*∗∗∗*^	−0.138	0.871	0.684	1.109	0.263
Valve	−0.044	0.957	0.773	1.186	0.690
CABG + valve	−0.114	0.892	0.707	1.125	0.333
Cardiopulmonary bypass time	0.012	1.012	1.010	1.013	<0.0001
Constant	−8.736	0.000			<0.0001

(d)					
Age	0.024	1.024	1.018	1.030	<0.0001
Gender	0.278	1.321	1.162	1.501	<0.0001
Sinus rhythm	−0.294	0.745	0.649	0.856	<0.0001
Creatinine	0.705	2.024	1.772	2.312	<0.0001
Reduced LV-EF^*∗*^	0.369	1.446	1.268	1.649	<0.0001
Severely reduced LV-EF^*∗∗*^	0.918	2.504	2.011	3.119	<0.0001
BMI category					
Cachexia	1.034	2.812	1.695	4.668	<0.0001
Obesity I	0.028	1.028	0.879	1.203	0.726
Obesity II	0.236	1.266	0.964	1.662	0.090
Obesity III	0.363	1.438	0.902	2.293	0.127
Comorbidities					
Diabetes mellitus	0.138	1.148	0.999	1.319	0.052
Arterial hypertension	0.135	1.145	0.985	1.330	0.079
Pulmonary obstruction	0.009	1.009	0.831	1.226	0.927
Pulmonal hypertension	0.233	1.262	1.087	1.465	0.002
Peripheral artery disease	0.151	1.163	0.943	1.433	0.157
Stroke in history	0.057	1.059	0.816	1.374	0.667
Malignancy	0.204	1.227	0.988	1.524	0.064
Introperative data					
CABG^*∗∗∗*^	−0.266	0.766	0.639	0.919	0.004
Valve	−0.291	0.748	0.634	0.882	0.001
CABG + valve	−0.334	0.716	0.595	0.862	<0.0001
Cardiopulmonary bypass time	0.010	1.010	1.009	1.012	<0.0001
Constant	−5.788	0.003			<0.0001

(e)					
Age	0.006	1.006	0.998	1.013	0.132
Gender	0.035	1.036	0.872	1.231	0.687
Sinus rhythm	−0.300	0.741	0.615	0.891	0.001
Creatinine	0.431	1.539	1.293	1.832	<0.0001
Reduced LV-EF^*∗*^	0.130	1.139	0.952	1.362	0.155
Severely reduced LV-EF^*∗∗*^	0.402	1.495	1.091	2.049	0.012
BMI category					
Cachexia	0.455	1.576	0.752	3.300	0.228
Obesity I	−0.021	0.979	0.794	1.208	0.843
Obesity II	−0.367	0.693	0.437	1.098	0.119
Obesity III	0.061	1.063	0.531	2.128	0.863
Comorbidities					
Diabetes mellitus	−0.054	0.948	0.779	1.153	0.592
Arterial hypertension	−0.068	0.934	0.776	1.125	0.472
Pulmonary obstruction	−0.253	0.776	0.578	1.042	0.092
Pulmonal hypertension	0.178	1.195	0.977	1.462	0.083
Peripheral artery disease	−0.127	0.881	0.644	1.205	0.426
Stroke in history	0.071	1.074	0.753	1.531	0.695
Malignancy	0.083	1.087	0.805	1.468	0.587
Introperative data					
CABG^*∗∗∗*^	−0.207	0.813	0.636	1.039	0.098
Valve	−0.180	0.835	0.671	1.039	0.105
CABG + valve	0.101	1.107	0.870	1.408	0.410
Cardiopulmonary bypass time	0.006	1.006	1.005	1.008	<0.0001
Constant	−4.151	0.016			<0.0001

(f)					
Age	0.021	1.021	1.011	1.031	<0.0001
Gender	0.224	1.251	1.022	1.531	0.030
Sinus rhythm	−0.062	0.940	0.750	1.176	0.586
Creatinine	0.618	1.855	1.541	2.233	<0.0001
Reduced LV-EF^*∗*^	0.112	1.118	0.900	1.390	0.313
Severely reduced LV-EF^*∗∗*^	0.550	1.733	1.212	2.477	0.003
BMI category					
Cachexia	0.613	1.846	0.836	4.078	0.130
Obesity I	−0.137	0.872	0.673	1.131	0.303
Obesity II	−0.370	0.691	0.399	1.196	0.186
Obesity III	−0.513	0.599	0.215	1.664	0.325
Comorbidities					
Diabetes mellitus	0.169	1.184	0.944	1.484	0.144
Arterial hypertension	−0.117	0.890	0.710	1.114	0.308
Pulmonary obstruction	−0.131	0.877	0.630	1.220	0.435
Pulmonal hypertension	−0.207	0.813	0.629	1.050	0.112
Peripheral artery disease	0.265	1.304	0.941	1.806	0.111
Stroke in history	0.836	2.307	1.679	3.170	<0.0001
Malignancy	0.140	1.150	0.817	1.620	0.423
Introperative data					
CABG^*∗∗∗*^	−0.853	0.426	0.319	0.569	<0.0001
Valve	−0.481	0.618	0.482	0.794	<0.0001
CABG + valve	−0.470	0.625	0.472	0.828	0.001
Cardiopulmonary bypass time	0.007	1.007	1.005	1.008	<0.0001
Constant	−5.701	0.003			<0.0001

^*∗*^Left ventricular ejection fraction: 31–50%; ^*∗∗*^Left ventricular ejection fraction ≤30%; ^*∗∗∗*^Coronary artery bypass grafting.

## Data Availability

The data used to support the findings of this study are available on request from the corresponding author.
